# Impaired inflammatory pain and thermal hyperalgesia in mice expressing neuron-specific dominant negative mitogen activated protein kinase kinase (MEK)

**DOI:** 10.1186/1744-8069-2-2

**Published:** 2006-01-16

**Authors:** Farzana Karim, Hui-Juan Hu, Hita Adwanikar, David Kaplan, Robert W Gereau

**Affiliations:** 1 Washington University Pain Center and Department of Anesthesiology, Washington University School of Medicine, 660 S. Euclid Ave, Campus Box 8054, St. Louis MO 63110, USA; 2 Department of Neuroscience and Cell Biology, University of Texas Medical Branch, Galveston, TX 77551, USA; 3 Department of Medical Genetics and Microbiology, University of Toronto and The Hospital for Sick Children, Toronto, ON, M5G 1X8, Canada; 4 Department of Anatomy and Neurobiology, Washington University School of Medicine, 660 S. Euclid Ave, St. Louis MO 63110, USA

## Abstract

**Background:**

Numerous studies have implicated spinal extracellular signal-regulated kinases (ERKs) as mediators of nociceptive plasticity. These studies have utilized pharmacological inhibition of MEK to demonstrate a role for ERK signaling in pain, but this approach cannot distinguish between effects of ERK in neuronal and non-neuronal cells. The present studies were undertaken to test the specific role of neuronal ERK in formalin-induced inflammatory pain. Dominant negative MEK (DN MEK) mutant mice in which MEK function is suppressed exclusively in neurons were tested in the formalin model of inflammatory pain.

**Results:**

Formalin-induced second phase spontaneous pain behaviors as well as thermal hyperalgesia measured 1 – 3 hours post-formalin were significantly reduced in the DN MEK mice when compared to their wild type littermate controls. In addition, spinal ERK phosphorylation following formalin injection was significantly reduced in the DN MEK mice. This was not due to a reduction of the number of unmyelinated fibers in the periphery, since these were almost double the number observed in wild type controls. Further examination of the effects of suppression of MEK function on a downstream target of ERK phosphorylation, the A-type potassium channel, showed that the ERK-dependent modulation of the A-type currents is significantly reduced in neurons from DN MEK mice compared to littermate wild type controls.

**Conclusion:**

Our results demonstrate that the neuronal MEK-ERK pathway is indeed an important intracellular cascade that is associated with formalin-induced inflammatory pain and thermal hyperalgesia.

## Background

Extracellular signal-regulated kinases (ERKs) belong to a cascade that is part of a phosphorelay system composed of three sequentially activated kinases regulated by phosphorylation. Initiation of this cascade occurs via multiple mechanisms which ultimately activate raf kinases. Activated raf phosphorylates MEK which phosphorylates ERK1 and ERK2 on tyrosine and threonine residues. Extracellular signal-regulated kinases are involved in the regulation of meiosis and mitosis, and in differentiated cells, ERKs integrate a wide variety of postmitotic functions [1-3]. Within the past decade, numerous studies in rodents have elucidated the role of ERKs in nociceptive plasticity. ERK activation is activity-dependent, and occurs following noxious stimulation [4,5]. The role of ERK in nociceptive plasticity has been extensively studied in the spinal cord and dorsal root ganglia, two important sites of nociceptive sensitization [6-9]. In addition to different types of noxious stimuli, high intensity electrical stimulation of C-fibers also activates ERK in the spinal cord dorsal horn, suggesting that C-fiber recruitment is crucial for release of transmitters that activate ERK centrally in the spinal cord [6,10].

ERK is expressed in neuronal as well as non-neuronal cells [11] and the above mentioned studies have shown that ERK activation occurs in both neuronal and glial cells of the spinal cord. A recent study showed that ERK is sequentially activated first in spinal neurons, then in microglia, and then in astrocytes during the development of neuropathic pain [12]. Activated microglia and astrocytes in the spinal cord play a pivotal role in mediating enhanced pain states. Noxious stimulation, such as occurs with a subcutaneous formalin injection in the paw, is associated with glial cell activation [13,14]. Inhibitors of microglial activation can reduce persistent pain states [15]. It is thought that glial cells may enhance pain states by releasing pro-inflammatory cytokines and other substances that facilitate pain transmission [16]. Because ERK has been shown to promote glial activation [17], it is possible that activation of ERK may lead to increased activity of spinal glial cells in persistent pain states. Taken together with previous studies showing that ERK is strongly activated in dorsal horn neurons in response to noxious stimuli, and ERK activation in dorsal horn neurons leads to alterations in K+ channel function and enhanced excitability of these cells [18,19], these data suggest that both neuronal and glial cells may contribute to enhanced pain transmission via ERK activation.

To study the importance of ERKs in nociception, most studies mentioned above have utilized intrathecal pharmacological inhibition of MEK using either PD 98059 or U0126, which may inhibit MEK function in both neuronal and non-neuronal cells. In addition to inhibiting ERK activation in multiple cell types, high doses of PD98059 have direct inhibitory effects on Cam Kinase II [20] and cyclooxygenase II [21]. U0126 used at higher doses, and particularly with continuous perfusions, may lead to motor effects [22] which may result in misinterpretation of withdrawal responses. To address the above concerns, and to evaluate the specific contribution of neuronal ERK activation to pain behavior, we aimed to test whether selective suppression of neuronal MEK activity can decrease nociceptive plasticity using the formalin model. We tested mutant mice that express dominant negative MEK, whose expression was driven by the pan-neuronal and neuron-specific Talpha1 alpha-tubulin promoter, such that the dominant negative MEK protein is expressed only in neurons. Our findings suggest that the neuronal MEK-ERK cascade is required for inflammatory pain plasticity.

## Results

### Reduced second phase of formalin test in the DN MEK mice

**Figure 1:**
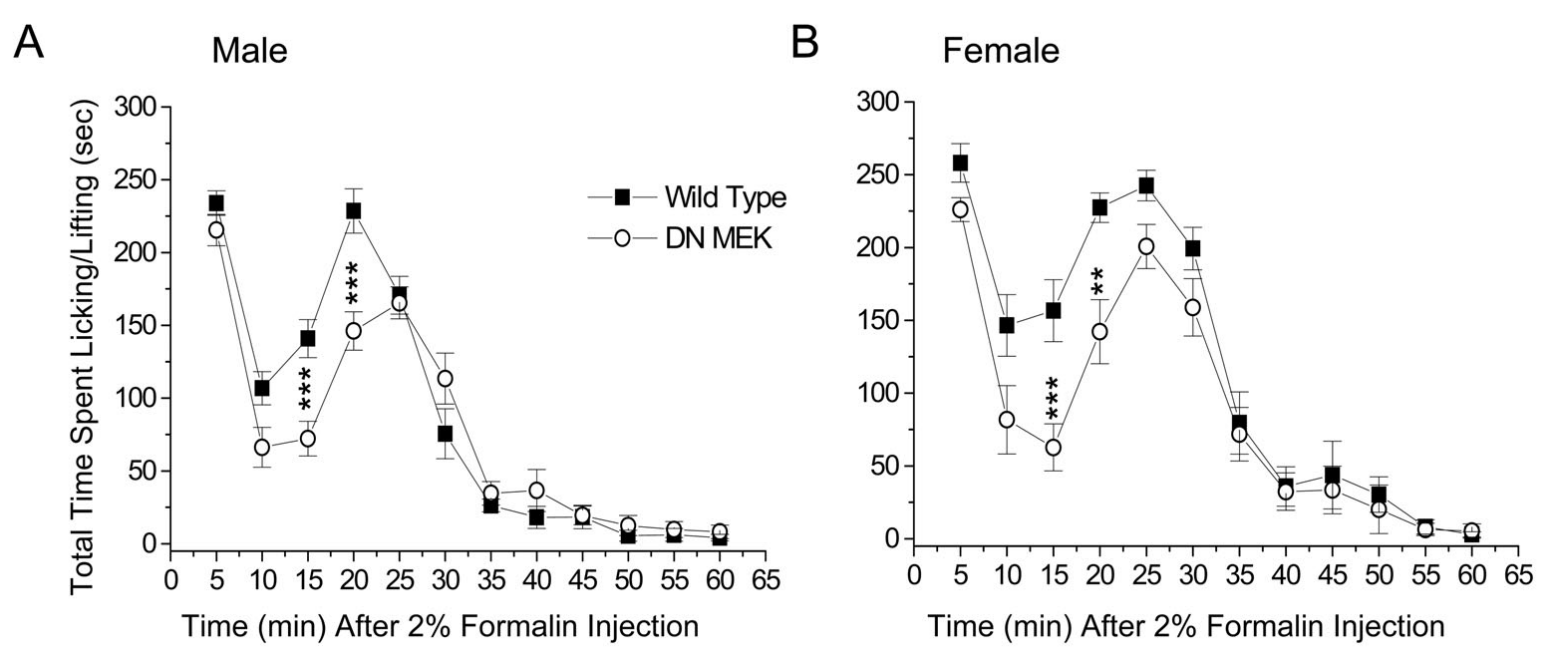
Reduced second phase of formalin-induced nociceptive behavior in DN MEK mice. Time course of nociceptive behavior following 2 % formalin injection in the hind paws of either male (A) or female (B) wild type or DN MEK littermate mice. n = 12–14 (male mice) per group, and 8–10 (female mice) per group. For this and the following graphs, data are presented as mean ± S.E.M. **p < 0.01, ***p < 0.001.

The formalin model is frequently used in the study of inflammatory pain states in rodents. Injection of 2 % formalin subcutaneously in the hind paw of mice results in a typical biphasic nociceptive response [23]. The first phase, which usually lasts less than 5 minutes, occurs a few seconds after formalin injection and is characterized by intense spontaneous licking or lifting of the injected paw. This phase is due to acute stimulation of nociceptors. The second phase is characterized by licking and lifting of the injected paw beginning at about 15–20 minutes after formalin injection and lasting until approximately 40–60 minutes after formalin. This second phase of nociception is thought to involve central sensitization of dorsal horn neurons as well as peripheral sensitization associated with the inflammation [24]. We previously showed that in mice, there is a reduced second phase of licking/lifting behavior following attenuation of ERK activity by intrathecal injection of the MEK inhibitor, PD 98059 [7]. In the present study, we investigated the effects of reduced neuronal MEK function in the DN MEK mice in the formalin test. We performed experiments in male and female mice since the MEK-ERK signaling pathway is a more dominant component of inflammatory hyperalgesia in females [25,26]. We find a significant gender difference in the response to formalin; the female mice of both groups have more significant spontaneous nociceptive behavior than the male mice (p < 0.001, ANOVA). The first phase was not altered in either the male or female DN MEK mice when compared to their wild type littermates. However, there was a significant reduction of the ascending part of the second phase of the formalin test in both male and female DN MEK mice (Fig 1). Thus, the neuronal MEK-ERK cascade is important for the development of the second phase of formalin-induced inflammatory nociception.

### Decreased thermal hyperalgesia in the DN MEK mice

**Figure 2:**
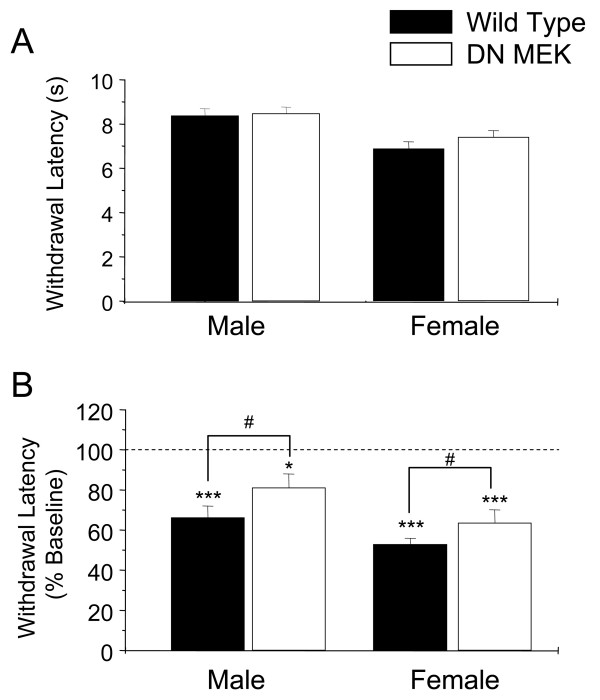
Reduced thermal hyperlagesia in DN MEK mice. A, Baseline thermal thresholds of male and female wild type and DN MEK mice. B, Thermal thresholds taken 1 to 3 hours following injection of 2 % formalin, expressed as % of baseline values (shown as 100%, dashed line). n = 11–16 mice per group (male mice), and 12–15 mice per group (female mice). *p < 0.05, ***p < 0.001, significant differences from baseline thresholds. #p < 0.05, significant differences between wild type and DN MEK mice.

We next investigated whether thermal hyperalgesia 1 to 3 hr after 2% formalin injection was altered in the DN MEK mice. Baseline withdrawal latencies to radiant heat determined before formalin injection were similar in wild type and DN MEK mice (Fig 2a). Female mice of both groups (wild type and DN MEK) express more thermal hyperalgesia compared to the male mice (P < 0.001). Both the wild type and DN MEK mice of both genders exhibited significant ipsilateral thermal hyperalgesia (Fig 2b); however, there is significantly less thermal hyperalgesia in the DN MEK mice measured 1–3 hours after formalin injection (Fig 2b) compared to their wild type littermates. These results show that the DN MEK mice have reduced inflammatory thermal hyperalgesia. The thermal thresholds of the contralateral uninjected paws were not significantly different from their baselines in both male and female mice (data not shown).

### Decreased thermal hyperalgesia following intrathecal U0126

**Figure 3:**
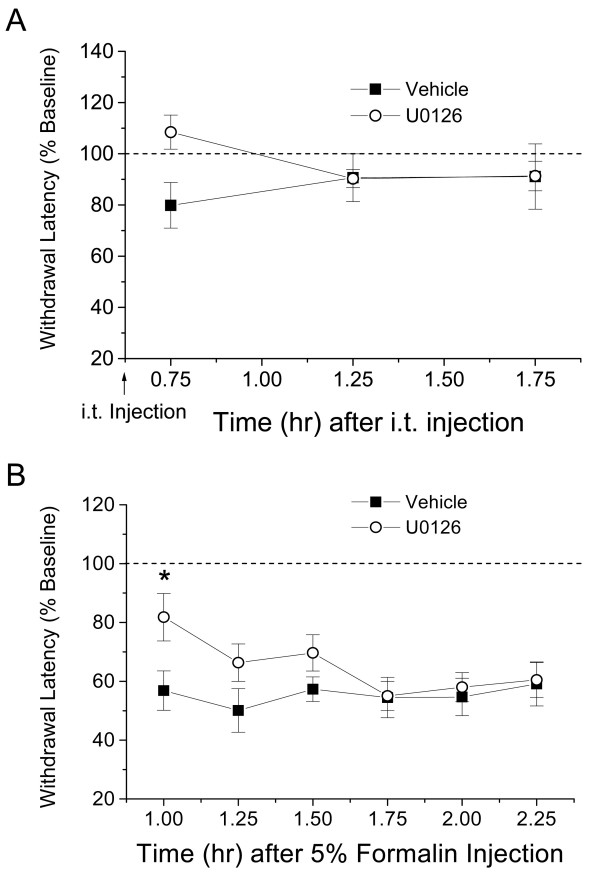
Intrathecal injection of the MEK inhibitor, U0126, reduces thermal hyperalgesia in wild type mice. A) Effect of intrathecal injection of vehicle (7.5 % DMSO in PBS, pH 7.4) or U0126 (2 nmols) on thermal thresholds in mice. B) Effect of intrathecal pretreatment of either vehicle or U0126 (2 nmols) 15 min prior to injection of 5 % formalin in the hind paw on thermal thresholds recorded 1 hr after formalin injection. n = 10 per group. *p < 0.05.

Following the observation that the DN MEK mice had less thermal hyperalgesia following formalin injection, we sought to determine whether the MEK inhibitor, U0126, injected intrathecally would reduce thermal hyperalgesia in wild type mice. A single intrathecal injection of U0126 (2 nmols, [22]) did not affect basal thermal thresholds (Fig 3a); however, this treatment significantly decreased thermal hyperalgesia 1 hour following formalin injection in the U0126 treated mice compared to their vehicle controls (Fig 3b).

### Reduced ERK activation after formalin in DN MEK mice

**Figure 4:**
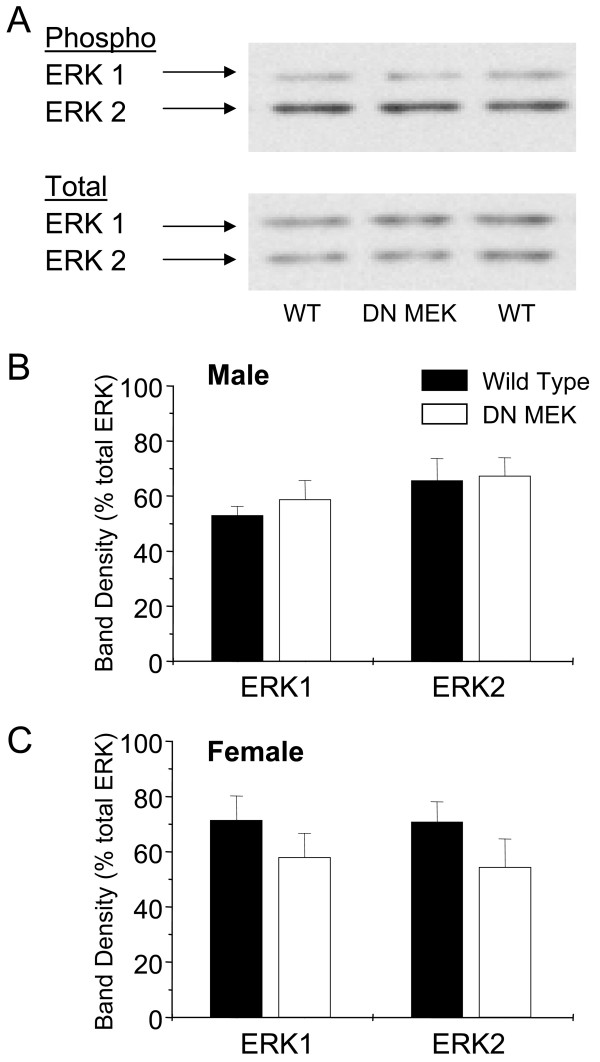
Basal phosphorylation of ERK is similar in the wild type and DN MEK mice spinal cords. A, Representative immunoblot of mouse spinal cord homogenates using a phospho-ERK1/2 antibody (top) or a total ERK1/2 antibody (bottom). The arrows show the position of the 44 kDa ERK 1 and 42 kDa ERK 2 isoforms. Quantification of ERK activation in male (B) and female (C) mice. Phospho-ERK bands were densitized and normalized to total ERK from the same samples. n = 8–9 (male mice), and 8–9 (female mice).

**Figure 5:**
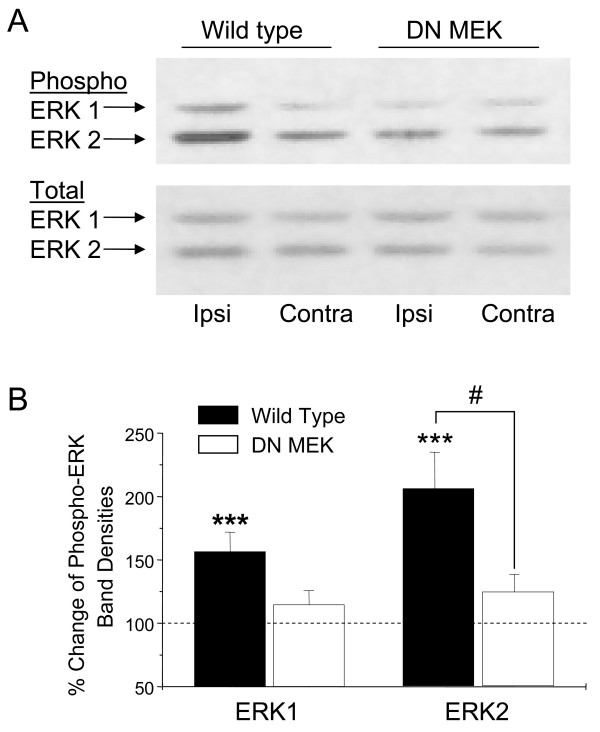
Reduced phospho-ERK in DN MEK mice spinal cords 15 minutes after 2 % formalin injection in the hind paw. A, Representative immunoblots of ipsilateral (ipsi) and contralateral (contra) mouse spinal cord homogenates from a wild type or a DN MEK mouse using a phospho-ERK 1/2 antibody (top) or a total ERK 1/2 antibody (bottom). B, Quantification of ERK1 and ERK2 bands. Phospho-ERK bands were densitized and normalized to total ERK bands and expressed as % change of phospho-ERK on the ipsilateral side compared to the contralateral side (100 %). ***p < 0.001, significant differences between ipsilateral phospho-ERK and contralateral phospho-ERK. #p < 0.05, significant differences in ipsilateral phospho-ERK between the wild type and DN MEK mice. n = 12 (wild type) and 7 (DN MEK).

We next investigated whether the reduced inflammatory nociception was associated with reduced ERK activation in the DN MEK mice. Extracellular signal-regulated kinases have a central role in nociceptive sensitization in the spinal cord; we therefore examined activation of ERK at this site in either wild type or DN MEK mice following formalin injection. Although basal phosphorylated ERK is minimal in mouse spinal cords without noxious stimuli, we investigated whether expression of the DN MEK transgene would alter basal ERK activation in the DN MEK mice. Levels of phosphorylated ERK in the lumbar spinal cords of naïve mice were determined by immunoblotting using a phospho-ERK-selective primary antibody. The phospho-ERK bands were quantified and normalized to total ERK immunoblotted from the same samples using an anti-total ERK1/2 antibody. There was no significant difference in the amount of basal phospho-ERK1 or phospho-ERK2 between the wild type and DN MEK mice spinal cords in either male or female mice (Fig 4a, 4b and 4c). We next investigated whether the DN MEK mice had reduced activation of ERK following formalin injection. We showed previously that injection of 2% – 5% formalin subcutaneously into the mouse hind paw induces a time-dependent activation of ERK in the lumbar spinal cord [7] which peaks at 3 minutes, remains sustained for up to 25 minutes and diminishes by 60 minutes. In the current experiment, mice were killed 15 minutes after 2 % formalin injection in the right hind paw. In the wild type mice, blots of tissue taken from the side of the spinal cord ipsilateral to the formalin injection showed significant stimulation of both ERK1 and ERK2 when compared to the contralateral side (Fig 5a and 5b), whereas ERK activation in the spinal cords from the DN MEK mice was not significantly different from their contralateral sides. Furthermore, ipsilateral ERK2 activation was significantly lower in the DN MEK mice than ipsilateral ERK2 activation in the wild type mice. Taken together, these results indicate that DN MEK mice have reduced formalin-induced inflammatory pain as well as reduced formalin-induced ERK activation in the spinal cord.

### Unmyelinated fiber counts in cross sections of the sciatic nerve

**Figure 6:**
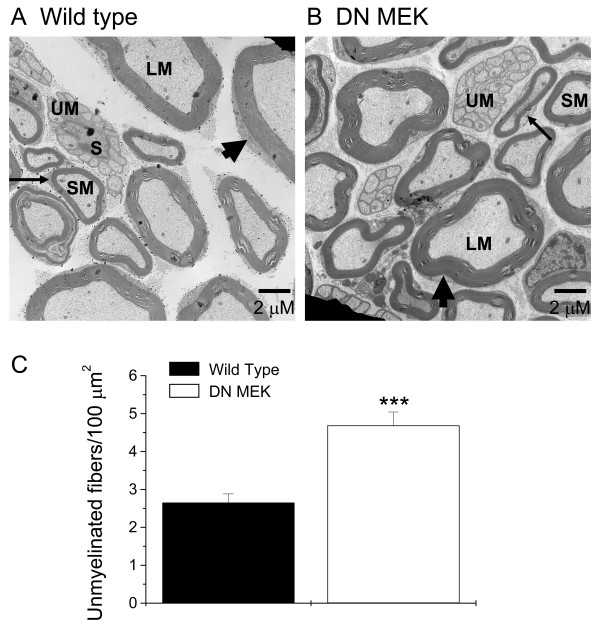
Representative electron micrographs of cross sections of the sciatic nerves of A) wild type and B) DN MEK mice. Small diameter unmyelinated fibers (UM) are often present as encapsulated bunches of fibers in between the myelinated fibers. Thick arrowheads point to the Schwann cells of the large diameter myelinated fibers (LM). Small arrows point to the Schwann cells of the small diameter myelinated fibers (SM). S represents the Schwann cell of the unmyelinated fibers. C) Bar graph showing the mean number of unmyelinated fibers per 100 μm^2^. ***P < 0.001, n = 363 and 377 images from 2 wild type and 2 DN MEK mice sciatic nerves respectively.

ERKs also play an important role in development [2]. Because recruitment of C-fibers is necessary for spinal ERK activation [6,10], we asked whether the reduction of spinal neuronal ERK activation might be due to a reduction in the number of peripheral unmyelinated fibers in the DN MEK mice. Electron microscopy of sciatic nerve sections of wild type or DN MEK mice revealed that the DN MEK mice had approximately twice the number of unmyelinated fibers as those counted in the wild type mice (Fig 6a, 6b, and 6c). Therefore, the reduction in spinal ERK activation in the DN MEK mice is not due to reduced number of unmyelinated peripheral fibers.

### Decreased ERK-mediated modulation of A-type potassium currents in DN MEK mice

**Figure 7:**
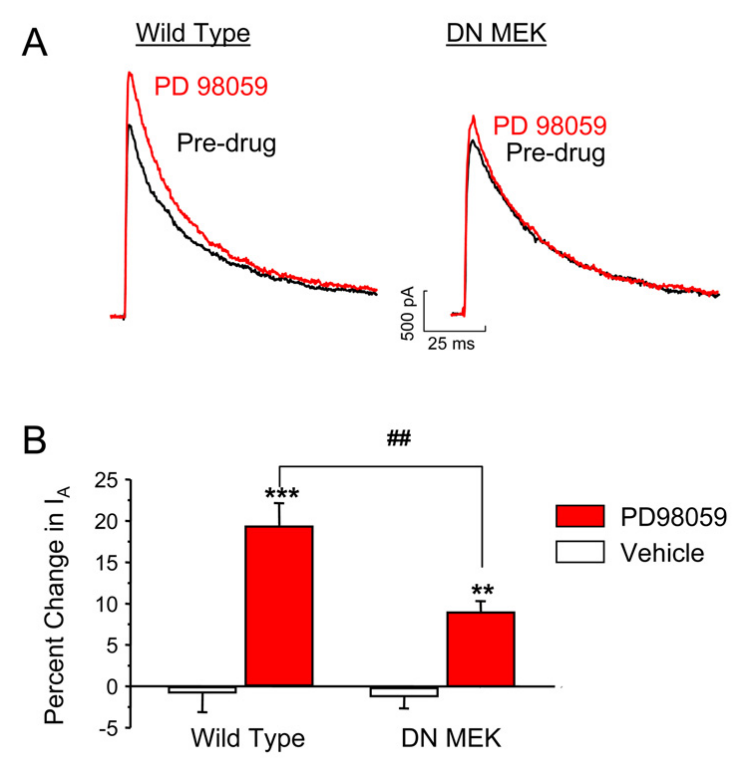
Reduced effect of the MEK inhibitor, PD 98059, on A-type potassium currents in spinal cord dorsal horn neuronal cultures from DN MEK mice. A) Representative examples of I_A _recorded before and after 5 minute bath application of 20 μM of PD 98059 (A-type currents were isolated from sustained currents as described in the methods section). B) Summary of percentage changes of peak amplitude of I_A _by PD 98059. n = 5 neurons from 4 mice per group. **p < 0.01, ***p < 0.001, significant differences from vehicle treatment. ^##^p < 0.01, significant differences between wild type and DN MEK mice.

To further investigate whether there is a functional deficit of the MEK-ERK cascade specifically in spinal cord neurons of the DN MEK mice, we asked whether ERK regulation of a downstream target, the transient A-type potassium channel, is altered in these mice. ERK is known to phosphorylate Kv4.2, an A-type potassium channel subunit [27], and we have previously shown that MEK inhibitors (U0126 or PD 98059) enhance A-type potassium currents in dorsal horn neurons of the spinal cord [19]. Dorsal horn cultures were prepared from either wild type or DN MEK mice, and the effect of bath application of 20 μM PD 98059 was examined. Neurons from the DN MEK mice were significantly less sensitive to modulation by the MEK inhibitor PD 98059 (Fig 7). These results confirm a reduced function of the MEK-ERK cascade in dorsal horn neurons from the DN MEK mice.

## Discussion

The present study reports several important findings regarding the role of the neuronal MEK-ERK cascade in nociception. The DN MEK mutant mice present a functional reduction of the activity of neuronal MEK, the kinase that selectively activates ERK 1 and ERK 2 [28]. The DN MEK mice have a reduced second phase of licking behavior following injection of 2% formalin in the hind paw compared to the responses of their wild type littermates. These data are in a sense similar to our previous pharmacological data where the intrathecally applied MEK inhibitor PD 98059, selectively reduced the second phase of licking behavior in mice [7]. However, the pattern of the second phase reduction is different between the pharmacological [6,7] and genetic suppression of neuronal ERK activation. PD 98059 provided a much stronger suppression of both the ascending and descending segments of the formalin second phase behavior. The current data shows a clear suppression, in both male and female mice, only during the ascending part of the second phase, suggesting that neuronal MEK-ERK cascade contributes to the development of the second phase spontaneous licking behavior. Perhaps the larger suppression induced by intrathecally applied MEK inhibitors is due to inhibition of both neuronal and non-neuronal ERK activation. Indeed it has been shown recently using a neuropathic model that ERK is sequentially activated first in neurons, followed by microglia, and later in astrocytes [12], and taken together with our present data, we suggest that neuronal ERK contributes to development of central sensitization, which may later be maintained by non-neuronal cells. Our data are also in agreement with a wealth of previous data reporting that MEK inhibitors reduce inflammatory pain using different pain models in rodents [6,29,30]. In the current study, we do not rule out the contribution of other nervous system structures to the reduced behavioral effect in the DN MEK mice. However, we do show that the contribution of the spinal cord to the reduced behavioral effect is paramount since the activation of ERK1 and ERK2 is also decreased following formalin injection in the DN MEK mice relative to wild type littermates, and the behavioral and biochemical inhibition can be mimicked by intrathecal administration of MEK inhibitors.

A recent paper reported decreased basal ERK activity in the hippocampi of the DN MEK mice [31]. In the current studies, we do not observe suppressed basal ERK activation in the spinal cords of the DN MEK mice. Basal ERK activation is minimal in the spinal cord and spinal ERK activation is activity-dependent and has been shown to occur upon noxious or electrical stimulation of the peripheral nerves [5-7]. It is unlikely that the decrease in basal hippocampal ERK activity could produce decreased nociception in the DN MEK mice. Shalin et al. (2004), showed that despite the deficits in contextual fear-conditioning in the DN MEK mice, these mice did not have sensory deficits but rather comparable activity and anxiety levels as that of the wild type mice. We show further in our study, that there are no differences in basal thermal thresholds.

Injection of 2% formalin in mice produced thermal hyperalgesia (measured 1–3 hours after formalin injection), and more so in female mice than in the male littermates. Ipsilateral thermal hyperalgesia was significantly reduced in both the female and male DN MEK mice when compared to littermate wild types. Parallel to these data, a single intrathecal injection of U0126 reduced thermal hyperalgesia induced by 2 % formalin in wild type mice. Reduction of thermal hyperalgesia in the DN MEK mice is possibly due to decreased central sensitization since we showed clearly that spinal ERK activation following formalin injection was decreased in these mice. Possible reduction of upstream activation of ERKs by glutamate through either NMDA receptors [6], group I metabotropic glutamate receptors [7,32] and/or neurotrophins such as BDNF [33-35] could decrease central sensitization processes leading to reduced thermal hyperalgesia. Although we do not rule out possible contributions of peripheral activation of ERK through activation of TRPV1 [6,36-40], this possibility seems unlikely because of the increased number of unmyelinated fibers in the DN MEK mice. However, future experiments will determine whether TRPV1 channels and/or their functions are altered in the DN MEK mice.

In this study we examined cross sections of the sciatic nerves of the DN MEK mice in order to determine whether reduced ERK activation following formalin injection was due to reduced number of unmyelinated peripheral fibers. We were surprised to observe more unmyelinated fibers in the sciatic nerves of the DN MEK mice, particularly given that these mice showed no change in baseline thermal sensitivity. However, similar unexpected findings have been reported in the literature. For example, mice overexpressing glial cell line-derived neurotrophic factor or nerve growth factor possess increased numbers of unmyelinated fibers, yet they do not display hyperalgesia [41]. The increased number of unmyelinated fibers in the DN MEK mice could be a result of reduced ERK activity during development. The MEK-ERK cascade has gained much attention recently regarding the role of these kinases in promoting neuronal cell death. Death of cerebellar granule neurons (CGN) cultured in low potassium concentrations is accompanied by persistent ERK activation [42]. Inhibition of persistent activation of ERK with either MEK inhibitors, or with overexpression of dominant negative MEK in the cultures, resulted in a decrease in cell death of the CGN [42,43]. Our present data from the DN MEK mice are the first *in vivo *data that support a novel and important role of the MEK-ERK cascade promoting neuronal survival in the whole animal. Future experiments will be designed to characterize this role in mice and especially how the presence of the DN MEK affects the development of primary afferent nerve fibers and their receptors in nociception.

The current studies further show that ERK-mediated modulation of A-type potassium channels is impaired in spinal dorsal horn neurons from DN MEK mice. ERKs are known to directly phosphorylate Kv4.2, a K+ channel alpha subunit that generates A-type potassium currents [27]. Reduced ERK modulation of A-type potassium channels may contribute to decreased central sensitization of spinal neurons leading to decreased pain after inflammation.

## Conclusion

We show here, using transgenic mice with reduced neuronal ERK activity, that neuronal ERK plays a key role in the development of inflammatory nociceptive behavior, and contributes to the processing of thermal hyperalgesia. Furthermore, our results suggest that A-type potassium channels may be possible downstream targets of ERKs in the regulation of inflammatory nociception.

## Materials and methods

### Tα1 DN-MEK transgenic mice generation

The generation of the DN MEK mice has been previously described [31]. Briefly, a 1.1 kb Tα1 α-tubulin promoter element that confers pan- neuronal and neuronal-specific expression of the transgene [44,45] was used to drive the expression of an HA-tagged K97M dominant negative form of MEK [31,46]. The transgenic mice were established in a C3H background strain, and back-crossed several generations with C57 Bl/6 mice. For genotyping, tail DNA was extracted following standard procedures and used for PCR analysis. The primers used to amplify the 436 bp HA-DN MEK transgene were sense 5'-CCC ATA CGA TGT TCC AGA TTA CGC-3' and antisense 5'-CGC ACC ATA GAA GCC CAC GAT G-3' (Sigma -Genosys).

### Animal behavioral nociceptive testing

All experiments were done in accordance with the Animal Care and Use Committee of Washington University School of Medicine. Mice were housed in 12 hr/12 hr light/dark cycles and given food *ad libitum*. Mice weighing 20–25 g were used for experiments. All experiments were done using littermate controls and were performed with the experimenter blind to the genotype. The formalin test was performed as described previously [7]. Mice were habituated in a transparent Plexiglas test box (5 × 5 × 10 inches) before any injections for I hr. 10 μl of 2 % formalin solution was injected subcutaneously into the right hind paw, and the mouse returned to the test box immediately. The total time spent in nociceptive behavior (licking and lifting of the injected paw) was recorded in blocks of five minutes for one hour. In separate experiments, mice were habituated in Plexiglas chambers for 2–3 hours, and baseline thermal thresholds recorded. 10 μl of 2 % formalin solution was injected subcutaneously into the right hind paw, and the mice were returned to the chambers. Thermal thresholds were measured 1 hr following injection of formalin, and recorded for up to 3 hours. Thermal thresholds were measured as the latency (seconds) to withdraw or lick the paw in response to a constant radiant heat source through the glass bottom of a chamber to the plantar surface of the hind paw (IITC Life Sciences, Woodland hills, CA) [47].

### Drug application

For electrophysiological recordings, the MEK inhibitor PD98059 (Sigma, St. Louis, MO) was dissolved in 100 % DMSO and diluted to the final concentration (20 μM) in HBSS (Invitrogen Life Technologies, Carlsbad, CA). PD 98059 was applied by perfusion continuously at approximately 2–3 ml/min. For behavioral experiments, U0126 (Biomol, Plymouth Meeting, PA) was first dissolved in 100 % DMSO and diluted with PBS, pH 7.4 to a final concentration of 2 nmols in 3 μl. U0126 or the final concentration of vehicle (7.5 % DMSO in PBS, Ph 7.4) was injected intrathecally (i.t.) in a volume of 3 μl by lumbar puncture using a Hamilton syringe and a 30 gauge needle.

### Sample preparation

Mice were sacrificed 15 minutes after hind paw formalin injection (2 %). The spinal cords were isolated and lumbar sections from individual mice were stored at -80°C. Lumbar spinal cord enlargements (L4-S1) where indicated, were separated into ipsilateral and contralateral sections and each homogenized using a dounce homogenizer in ice-cold homogenization buffer (50 mM Tris HCl, pH 7.5; 50 mM NaCl; 10 mM EGTA; 5 mM EDTA; 2 mM sodium pyrophosphate, 1 mM sodium orthovanadate; paranitrophenylphosphate; 1 mM phenylmethylsulfonyl fluoride, 20 μg/ml leupeptin and 4 μg/ml aprotinin, Sigma-Aldrich, St. Louis, MO). Protein concentrations were determined by the DC assay kit (Bio-Rad laboratories, Hercules, CA).

### Immunoblotting for total and phospho-ERK

10 μg of total protein was electrophoresed in 10% SDS polyacrylamide gels. Proteins were transferred onto protein sensitive nitrocellulose membranes and blocked in B-TTBS (3% bovine serum albumin (BSA); 50 mM Tris-HCl pH 7.5; 150 NaCl; 0.02 mM Na Orthovanadate; 0.05% Tween 20; 0.01% Thimerosal, Sigma-Aldrich, St. Louis, MO) for 1 hour at room temperature. All antibody applications were done in B-TTBS. An antiphospho-p44/42 ERK primary antibody that detects ERK phosphorylation at both Thr202 and Tyr204 (1:1000 dilution in B-TTBS, Cell Signaling Technology, Beverly, MA), and an Anti-p44/42 ERK primary antibody (1:1000 dilution in 3% BSA, Cell Signaling Technology, Beverly, MA) that detects total p44/42 isoforms were used for immunoblotting overnight at 4°C. The blots were washed and incubated in HRP-conjugated secondary antibody for 1 hour at room temperature. Blots were developed with enhanced chemiluminescence (ECL, Amersham, Arlington Heights, IL). Densitometric quantification of immunopositive bands for total or phospho-ERK 1/2 were performed using Scion Image software.

### Cell culture

All reagents for cell culture were purchased from Invitrogen Life Technologies, Carlsbad, CA, except where otherwise mentioned. Primary cultures of spinal cord dorsal horn were prepared from 3–7 day old mice using our previous protocol [19]. Briefly, the mice were killed by decapitation and a laminectomy performed to obtain the spinal cord. The spinal cord superficial dorsal horn was isolated and chopped into several strips which were incubated for 45 minutes at 37°C in Hank's balanced salt solution (HBSS) containing papain (15 U/ml: Worthington Biochemical, Lakewood, NJ). The strips were rinsed 3 times with HBSS, and placed in culture medium containing Neurobasal, 5% fetal calf serum, 5% heat-inactivated horse serum, 100 U/ml penicillin, 100 μg/ml streptomycin, 2 mM L-glutamax-1, 1% B-27 and 12 mM glucose (SIGMA-ALDRICH, St. Louis, MO). The cells were dissociated by triturition with a fire-polished Pasteur pipette. The cells were plated onto poly-D-lysine and collagen-coated coverslips, and cultured for 1 to 2 days in humidified air with 5% CO_2 _at 37°C.

### Electrophysiological recording

Whole cell recordings were performed as described in our previous work [19]. Briefly, whole cell recordings were made by standard procedures at room temperature with an EPC-10 amplifier and PULSE software (HEKA Elektronic, Lambrecht, Germany). Electrodes were pulled from filamented borosilicate glass and fire polished to a resistance of 3–6 MΩ. Most neurons had series resistance around 6–10 MΩ (range, 5–18 MΩ), which was compensated ≥65%. Input resistance was 1.00 ± 0.05 GΩ. Most neurons had leak currents < 100pA (-80 mV) which were not subtracted on-line. The bath solution (HBSS) contained 500 nM TTX and 2 mM CoCl_2 _to block voltage gated Na+ currents, Ca^2+ ^currents and Ca^2+ ^-activated K^+ ^currents. The electrode solution contained (in mM): 140 KCl, 1 mgCl_2_, 0.5 CaCl_2_, 5 EGTA, 10 HEPES, 3 Na_2_ATP, 0.3 Na_2_GTP, pH adjusted to 7.4 with KOH. The membrane voltage was held at -80 mV and potassium currents were evoked by a command potential of +40 mV. The transient A-type current was isolated by subtracting the sustained current evoked by a step to +40 mV with a 150 ms prepulse to -10 mV [19].

### Unmyelinated fiber counts in cross sections of the sciatic nerve

Mice were anesthetized with 50 mg/kg pentobarbital and the skin on the dorsal thigh was cut open. The muscles were separated with blunt dissection and the sciatic nerve exposed. One centimeter of the nerve was removed and immersed in a fixative (CAC) containing 2% paraformaldehyde, 2.5% gluteraldehyde, 0.1 M cacodylic acid pH 7.2 for 1 hour. After multiple rinses in CAC, samples were then fixed in 1% osmium tetroxide in CAC for an additional hour and then stained enblock in 1% uranyl acetate in h20 for 1 hour. Samples were then dehydrated through a series of ETOH, propylene oxide and then infiltrated and embedded in monomeric Embed 812 (Electron Microscopy Sciences). Blocks were sectioned with an RMC MTXL ultramicrotome at approximately 75–80 nm with a Diatome diamond knife, stained with Pb citrate, Uranyl acetate. Grids were viewed on a Hitachi H7500 transmission electron microscope with Advanced Microscopy Techniques (AMT) digital image capturing software and Hamamatsu digital camera. A total of 363 and 377 micrographs, from 2 wild type and 2 DN MEK mice respectively, were captured diagonally across the sciatic nerve by a person blind to the genotypes, and the images saved. The number of unmyelinated fibers was counted and the mean number of unmyelinated fibers was expressed per 100 μm^2^.

### Statistical analysis

Behavioral and immunoblot data were analyzed using one way or two way ANOVA on the Prism statistical program, followed by Bonferroni's or Newman-Keuls post hoc tests. Data from electrophysiology was evaluated off-line using Pulsefit software (HEKA Elektronic, Lambrecht, Germany) and Origin (Microcal Software, Northampton, MA) and Paired Student's t-test was used to compare drug effects from controls. Unpaired Student's t-test was used to compare the number of unmyelinated fibers between the DN MEK and wild type mice.

## Competing interests

The author(s) declare that they have no competing interests.

## Authors' contributions

FK performed the behavior, biochemical and anatomical studies and drafted the manuscript. HH performed the electrophysiological studies. HA participated in unpublished experiments and in drafting the manuscript. DK provided the transgenic mice for the studies, and helped to draft the manuscript. RWG conceived of the study, participated in its design and its coordination, and helped in drafting the manuscript. All authors read and approved the final manuscript.
